# Cardiopulmonary performance in allogeneic hematopoietic cell transplantation recipients—evaluation of pre-transplant risk assessments

**DOI:** 10.1038/s41409-020-01191-9

**Published:** 2021-01-06

**Authors:** Antonia Pahl, Sarah Waibel, Anja Wehrle, Gabriele Ihorst, Albert Gollhofer, Hartmut Bertz

**Affiliations:** 1 Department of Medicine I, Faculty of Medicine and Medical Center, University of Freiburg, Freiburg, Germany; 2 Department of Neurology and Neuroscience, Faculty of Medicine and Medical Center, University of Freiburg, Freiburg, Germany; 3 Department of Sport and Sport Science, University of Freiburg, Freiburg, Germany; 4 Clinical Trials Unit, Faculty of Medicine and Medical Center, University of Freiburg, Freiburg, Germany

**Keywords:** Disease-free survival, Haematological cancer, Quality of life

## Abstract

Cardiopulmonary performance reflects how well different organ systems interact. It is inter alia influenced by body composition, determines patients’ quality of life and can also predict mortality. However, it is not yet used for risk prediction prior to allogeneic hematopoietic cell transplantations (alloHCT). Thus, we aimed to examine the predictive power of peak oxygen consumption (VO2peak) as a representative of cardiopulmonary performance and that of body composition before alloHCT to determine overall survival (OS) and non-relapse mortality (NRM) 2 years after transplantation. We also compared it with the predictive power of four commonly-used risk scores: revised Pretransplant Assessment of Mortality (rPAM), Hematopoietic Cell Transplantation-specific Comorbidity Index (HCT-CI), revised Disease Risk Index (rDRI), European Society for Blood and Marrow Transplantation (EBMT). Fifty-nine patients performed a cardiopulmonary exercise test and body composition assessments before alloHCT and were observed for 2 years. Sixteen patients died. VO2peak and most risk scores assessed pre-transplant revealed no association with OS or NRM. Body composition parameters only within univariable analyses. But higher rDRI and the male sex, were associated with shorter OS and higher NRM. We thus propose that the current risk assessments be reconsidered. The predictive value of VO2peak and body composition need further clarification, however.

## Introduction

In recent decades, the number of allogeneic hematopoietic cell transplantations (alloHCT) has risen [1] and due to the use of reduced intensity conditioning protocols, alloHCT can be offered to older patients and those with more comorbidities [2, 3]. Pre-transplant assessments enable an individual risk-benefit ratio, as all-cause mortality still amounts to 46% [3]. In this context, calculating individual risk scores to predict overall survival (OS) and non-relapse mortality (NRM) are used prior to alloHCT. Commonly used scores are [4, 5] the European Blood and Bone Marrow Transplantation (EBMT) score [6], the Hematopoietic Cell Transplantation-Comorbidity-Index (HCT-CI) [7] the revised Disease Risk Index (rDRI) [8] and the revised Pre-transplant Assessment of Mortality score (rPAM) [9]. As none of these scores covers all important variables, the concurrent use of various scores is recommended to include various aspects of risk prediction. However, according to an analysis by Shouval et al. [5] the forecast reliability of those scores ranges from moderate to random. To specify the individual risk-benefit ratio and provide an additional basis for medical decisions, e.g., the intensity of conditioning therapy, patients’ organ functions are usually determined by medical tests. The body mass index (BMI) is also calculated. Although muscle weakness and restrictions of the cardiorespiratory system are common therapy related side effects [10–13] that may cause mortality [14, 15], so far the level of physical performance is not studied in this context. Stress tests could therefore help us estimate the interaction of different organ’s function and allow more accurate assessment of patients’ physical performance. A cardiopulmonary exercise test (CPET) covers pulmonary, cardiovascular, blood circulation, and muscle systems’ interaction, thereby providing a comprehensive evaluation of a patient’s physical performance and organ function under stress. Peak oxygen intake (VO2peak) as a main CPET outcome is known to predict mortality in healthy individuals [16] and in breast or colon cancer survivors [17, 18]. There are initial indications that pre-alloHCT VO2peak could also provide information for predicting OS and NRM [19, 20]. But these results have not yet been confirmed or compared to existing risk calculation methods. CPET outcomes are also influenced by patients’ body composition parameters [21, 22]; the phase angle in particular also predicts clinical outcomes [23, 24]. However, the validity of these individual risk prediction methods—the calculation and use of risk scores, examining physical functioning and body composition parameters—have not been concurrently verified within one sample.

Aim of the present analysis was therefore to examine the explanatory power of pre-alloHCT VO2peak and body composition to predict OS and NRM 2 years after transplantation. We also compared this risk prediction to those of commonly-used risk scores. We hypothesized that assessing VO2peak and body composition would improve the present risk prediction methods and thus enable a more accurate post-alloHCT prognosis.

## Materials and methods

### Study design and patients

This survival analysis relied on the baseline data from a randomized controlled trial [25]. This study was designed to investigate the effects of whole body vibration training on patient’s physical performance during alloHCT. Examinations were performed before conditioning therapy, at hospital discharge and 180 days after alloHCT. Within a 17-month period, patients scheduled for alloHCT were consecutively recruited at the Department of Medicine I, University Medical Center Freiburg, Germany on the day of hospital admission. Included patients underwent study assessments prior to their first administration of conditioning therapy including CPET and body composition analysis in the course of baseline procedure. More information about the study design and inclusion and exclusion criteria can be found elsewhere [25]. For OS- and NRM-analysis, patients were followed for 2 years after alloHCT. Only patients with hematological malignancies were included in the survival analysis.

### Cardiopulmonary exercise test

VO2peak was examined during CPET. Patients performed an incremental CPET on an electronically-braked cycle ergometer (ergoline ergoselect 1200, Ergoline GmbH, Bitz, Germany) in recumbent (40°) position during continuous monitoring of their electrocardiogram, heart rate, and blood pressure. Gas exchange and ventilation were recorded continuously via breath-by-breath gas analysis (MetaLyzer 3B-R3, Cortex Biophysik GmbH, Leipzig, Germany). Patients had to perform until exhaustion starting at 20 W with a 10-W increase every minute. Exhaustion was determined by respiratory exchange ratio (RER) ≥ 1.1 or a heart rate ≥85% of the age predicted maximum [26, 27]. The RER describes the ratio of carbon dioxide produced and oxygen consumed. It is an indicator of metabolic fuel and therefore rates patients metabolic effort [21]. VO2peak describes the maximum amount of oxygen uptake during CPET, and is influenced by pulmonary, cardiovascular, hematopoietic, neuropsychological and muscular functioning and interaction [21, 28]. We also documented the data on maximum reached watt (Pmax) and received perception exertion (RPE). RPE also reflects patients’ effort rated subjectively on a scale from 6 to 20 immediately upon discontinuing CPET [29].

### Body composition

BMI was calculated as body weight (kg) divided by squared height (m²). Bioelectrical impedance analysis was used to determine body fat mass (FM) (%), lean body mass (LBM) (%), body cell mass (BCM) (%), and phase angle (°) (BIA, Nutriguard-S, Data Input, Pöcking, Germany). This measurement can reveal cell membrane function, and thus evaluates body composition in more detail [30]. We applied sex-, age-, and BMI-specific references [31] to calculate the standardized phase angle according to [23]: $\frac{{{\mathrm{phase}}\;{\mathrm{angle}} - {\mathrm{references}}}}{{{\mathrm{standard}}\;{\mathrm{deviation}}\;{\mathrm{references}}}}$. To ensure better validity, all measurements were taken in the morning after at least 20 min rest, in lying position and before breakfast [32].

### Risk scores and clinical parameters

We calculated the EBMT score and HCT-CI prospectively before alloHCT, whereas the rPAM score and the rDRI were calculated retrospectively for this analysis. Information on the contents of the individual scores, their range and the classification of the risk groups can be found in Table 1. For our analysis we have combined the low and intermediate risk group of the rDRI. Information on OS and NRM was collected during medical follow-up examinations. Medical characteristics and history were retrospectively extracted from medical reports.

**Table 1:** Pre-alloHCT risk scores.

Score	Contents	Range	Groups
EBMT [6, 33]*	Patients age, Disease stage, Donor type, Gender constellation: donor–recipient	0–7	Low risk (0–2)Intermediate risk (3–5) High risk (6, 7)
HCT-CI [7]*	Secondary diagnosis and restrictions of organ function	0–29	Low risk (0) Intermediate risk (1,2) High risk (≥3)
rDRI [8]*	Disease, Disease stage, Cytogenetics	n.a.	Low risk Intermediate risk High risk Very high risk
rPAM [9]*	Patient age, Disease risk, Donor type, FEV1, CMV–constellation: donor-recipient	0–41	n.a.

*EBMT* European Society for Blood and Marrow Transplantation; *HCT-CI* Hematopoietic Cell Transplantation-Specific Comorbidity Index; *rDRI* Revised Disease Risk Index; *rPAM* Revised Pretransplant Assessment of Mortality; *n.a.* not available; *FEV1* forced expiratory pressure in 1 second; *CMV* cytomegalovirus

*References.

### Quality of life

We used the subscales “global quality of life (QoL)” and “physical function” of the EORTC QLQ-C30-questionnaire to supplement description of patients’ physical functioning and for survival analysis.

### Statistical analyses

Patients’ characteristics and physical conditioning were described using absolute and relative frequencies or median (range) respectively. Since all patient data were observed over exactly 24 months, we evaluated differences between 2-year-survivors (2yrS) and 2-year-decedents (2yrD) via nonparametric tests. Relapse was considered as a competing risk. Patients still alive after two years of observation were censored at this time. The Kaplan–Meier method was used to estimate the probability of survival. Cox’s method was used for univariable and multivariable analyses on OS. Analyses on NRM were done by Fine & Gray model. Results are presented as hazard ratios (HR) for Cox’s model and sub-distribution HR (SHR) for the Fine & Gray model, together with two sided 95% confidence intervals (CI). For continuous prognostic variables, the HR/SHR describes the risk increase for a one unit (or unit specified in the table) increase in the prognostic variable. The variable selection for multivariable models is based on profound theoretical considerations, including univariable results and possible correlations between prognostic factors to reduce multicollinearity. Gender, remission, conditioning, the EBMT score, rDRI and HCT-CI were examined as categorical variables. The Karnofsky Performance Scale (KPS) was categorized in below and above or equal 90%. All other parameters were calculated as continuous variables. The proportional hazards assumption was tested using graphic methods. Statistical analyses were conducted using the IBM SPSS Version 25 (SPSS Inc., Chicago, Illinois, USA) and SAS 9.4 (SAS Institute Inc., Cary, NC, USA) software.

## Results

We enrolled a sample of 71 patients in this study [25]. Data on 59 patients were used for survival analysis: four patients had to be excluded due to a nonmalignant disease, two died before alloHCT, medical data were missing on one patient, and five patients were unable to perform CPET (four patients due to inappropriate blood values, one due to a respiratory infection). All patients received peripheral blood stem cells from an unrelated donor. Patients’ characteristics is presented in Table 2.

**Table 2:** Patient characteristics.

	All *N* = 59	2yrS *N* = 43	2yrD *N* = 16	*P* value
Age [years]^a^	56 (19–78)	54 (19–75)	61 (31–78)	0.063
Sex [*n*]^b^				
Male:female	40:19 (68:32)	27:16 (63:37)	13:3 (81:19)	
Disease [*n*]^b^				0.715
Myeloid^c^	46 (78)	34 (79)	12 (75)	
Lymphatic^d^	15 (22)	9 (21)	4 (25)	
Remission at alloHCT [*n*]^b^				0.591
CR/chronic phase	27 (46)	23 (54)	4 (25)	
Not in CR	32 (54)	20 (46)	12 (75)	
HLA-mismatch [*n*]^b^				0.650
None (10/10)	36 (61)	27 (62.8)	9 (56.3)	
Mismatch (≤9/10)	23 (39)	16 (37.2)	7 (43.8)	
Gender donor: recipient				0.964
Female:male	15 (25)	11 (26)	4 (25)	
All other	44 (75)	32 (74)	12 (75)	
Conditioning [*n*]^b^				0.168
Myeloablative	15 (25.4)	12 (27.9)	3 (18.8)	
* TT/BU/FLU*	*12 (20.3)*	*9 (21)*	*3 (18.8)*	
* TBI/VP16*	*1 (1.7)*	*1 (2.3)*	*0*	
* TBI/TT*	*1 (1.7)*	*1 (2.3)*	*0*	
* FLU/BU 4*	*1 (1.7)*	*1 (2.3)*	*0*	
Reduced intensity	44 (74.6)	31 (72.1)	13 (81.2)	
* FLU/BCNU/MEL*	*14 (23.7)*	*10 (23.3)*	*4 (25)*	
* FLU/BCNU/TT*	*1 (1.7)*	*1 (2.3)*	*0*	
* FLU/TT/MEL*	*28 (47.5)*	*20 (46.5)*	*8 (50)*	
* FLU/TT mod.* *+* *TREOS*	*1 (1.7)*	*0*	*1 (6.2)*	
Time diagnosis to alloHCT [days]^a^	214 (51–2569)	187 (51–2569)	550 (85–1702)	0.068
KPS [%]^a^	90 (70–100)	90 (70–100)	90 (70–100)	0.375
HCT-CI [*n*]^b^				0.550
Low risk (0)	17 (29)	13 (30)	4 (25)	
Intermediate risk (1–2)	20 (34)	15 (35)	5 (31)	
High risk (≥3)	22 (37)	15 (35)	7 (44)	
EBMT score [*n*]^b^				0.768
Low risk (0–2)	7 (12)	5 (12)	2 (13)	
Intermediate risk (3–5)	36 (61)	27 (63)	9 (56)	
High risk (6–7)	16 (27)	11 (25)	5 (31)	
rDRI [*n*]^b^				0.139
Low risk	1 (1.7)	1 (2.3)	0	
Intermediate risk	32 (54.2)	24 (55.8)	8 (50)	
High risk	18 (30.5)	16 (37.2)	2 (12.5)	
Very high risk	8 (13.6)	2 (4.7)	6 (37.5)	
rPAM score [score]^a^	23.9 (12.1–42.1)	22.9 (12.1–33.5)	25 (16.4–42.1)	0.192
Acute GVHD [*n*]^b^				0.687
None	29 (49.2)	21 (48.8)	8 (50)	
Grade I	11 (18.6)	9 (20.9)	2 (12.5)	
Grade II	6 (10.2)	6 (14)	0	
Grade III	11 (18.6)	5 (11.6)	6 (37.5)	
Grade IV	2 (3.4)	2 (4.7)	0	
Chronic GVHD [*n*]^b^				0.022^*^
None	32 (54.2)	24 (55.8)	8 (50)	
Limited	8 (13.6)	8 (18.6)	0	
Extensive	11 (18.6)	11 (25.6)	0	
Missing data^e^	8 (13.6)	0	8 (50)	
Causes of death [*n*]^b^				
Alive	43 (72.9)	43 (100)	0	
Relapse/progression disease	4 (6.8)	0	4 (25)	
Organ failure	1 (1.7	0	1 (6.3)	
GVHD	5 (8.5)	0	5 (31.3)	
Infection	6 (10.2)	0	6 (37.5)	

*2yrsS* 2-year survivor, *2yrsD* 2-year decedents, *HLA* human leukocyte antigen, *TT* thiotepa, *BU* busulfan, *FLU* fludarabine, *TBI* total body irradiation, *VP16* etoposide, *BCNU* camustin, *MEL* melphalan, *TREOS* treosulfan, *ALL* acute lymphocytic leukemia, *CR* complete remission, *KPS* Karnofsky Performance Scale, *HCT-CI* Hematopoietic Cell Transplantation-specific Comorbidity Index, *EBMT* European Society for Blood and Marrow Transplantation, *rDRI* revised Disease Risk Index, *rPAM* revised Pretransplant Assessment of Mortality, *GVHD* graft-versus-host disease.

^a^Median (range).

^b^percentage of *N* (%).

^c^Includes: acute myeloid leukemia, chronic myeloid leukemia, chronicle myelomonocytic leukemia, osteomyelofibrosis and myelodysplastic syndrome.

^d^Includes: hodgkin disease, non-hodgkin lymphoma, multiple myeloma, chronic lymphatic leukemia, acute lymphatic leukemia.

^e^Patients had died by the time chronic GVHD can occur (100 days after alloHCT).

### Clinical outcomes

**Fig. 1:**
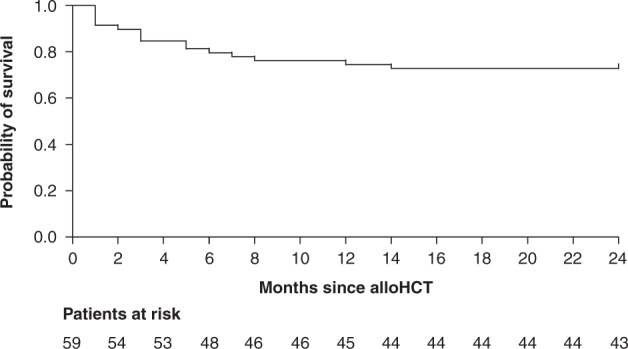
Kaplan–Meier curve presenting overall survival of 59 analyzed patients. Additionally patients at risk are presented in a two-month interval.

During the observation period (24 months) 16 of 59 patients died. The median survival time of deceased patients was 3.3 months (0.6–14 months). Forty-three patients were still alive and thus censored after 2 years. Median OS (50% of patients died) was not reached (Fig. 1). Univariable and multivariable analyses showed that VO2peak was not associated with either OS (Table 3) or NRM (Table 4). Same results are shown for three of four risk scores, age, KPS, BMI, phase angle, and total energy consumption. However, the rDRI, FM, LBM, and BCM revealed associations with OS and NRM in univariable analyses (rDRI: high risk HR 0.184, 95% CI 0.062–0.544, *p* = 0.002; very high risk HR 0.078, 95% CI 0.015–0.395, *p* = 0.002, SHR 0.076, 95% CI 0.009–0.641, *p* = 0.018; FM: HR 0.974, 95% CI 0.954–0.995, *p* = 0.014; SHR 0.974, 95% CI 0.965–0.983, *p* ≤ 0.001; LBM: HR 0.977, 95% CI 0.956–0.997, *p* = 0.026; SHR 0.976, 95% CI 0.967–0.985, *p* ≤ 0.001; BCM: HR 0.975, 95% CI 0.955–0.996, *p* = 0.021, SHR 0.975, 95% CI 0.966–0.984, *p* ≤ 0.001). Remission was only associated with OS (HR 0.315, 95% CI 0.101–0.979, *p* = 0.049). Multivariable models confirmed the significant association between rDRI and OS (very high risk HR 18.007, 95% CI 3.878–83.602, *p* ≤ 0.001) and NRM (high risk SHR 0.088, 95% CI 0.027–0.281, *p* ≤ 0.001; very high risk SHR 0.018, 95% CI 0.003–0.098, *p* ≤ 0.001). Patients in the very high risk group had a 18 times higher mortality risk than those of the low/ intermediate risk group. The male sex also increases overall mortality by a factor of six (HR 6.668, 95% CI, 1.440–30.882, *p* = 0.015).

**Table 3:** Cox regression analyses—association of pre-transplant variables with overall survival.

	HR	CI 95%		*P* value
		Lower	Upper	
Univariable analyses				
*VO2peak* [ml/min/kg]	0.937	0.628	1.400	0.752
*Gender*^a^	0.457	0.130	1.606	0.222
*Age* [years]	1.030	0.991	1.072	0.137
*Remission*^b^	0.315	0.101	0.979	0.046^*^
*Conditioning*^c^	0.491	0.112	2.161	0.347
rPAM score [score]	1.082	0.996	1.176	0.063
HCT-CI^d^				
Intermediate risk	0.673	0.197	2.301	0.528
High risk	0.762	0.242	2.400	0.642
EBMT score^d^				
Intermediate risk	0.898	0.174	4.630	0.897
High risk	0.773	0.259	2.307	0.644
rDRI^e^				
High risk	0.184	0.062	0.544	0.002^**^
Very high risk	0.078	0.015	0.395	0.002^**^
KPS < 90%	1.452	0.468	4.507	0.519
*BMI* [kg/m²]	0.968	0.853	1.099	0.619
Fat Mass [%]^10^	0.974	0.954	0.995	0.014^*^
Lean Body Mass [%]^10^	0.977	0.956	0.997	0.026^*^
Body Cell Mass [%]^10^	0.975	0.954	0.996	0.020^*^
Phase angle standardized [°]^f^	0.980	0.610	1.575	0.935
Total energy consumption during exercises/week [kcal]	0.993	0.982	1.005	0.238
Quality of life [%]	1.013	0.985	1.041	0.370
Physical functioning [%]	1.003	0.975	1.031	0.851
Multivariable analysis				
VO2peak [ml/min/kg]^5^	1.090	0.670	1.773	0.729
Gender^a^	6.668	1.440	30.882	0.015^*^
BMI [kg/m²]	0.873	0.727	1.050	0.149
Body cell mass [%]^10^	1.010	0.981	1.039	0.496
rDRI^e^				
High risk	0.526	0.095	2.908	0.461
Very high risk	18.007	3.878	83.602	<0.001^**^
Conditioning^c^	0.272	0.056	1.320	0.106

*HR* hazard ratio, *CI* confidence interval, *VO2peak* peak oxygen consumption, *rPAM* revised pretransplant assessment of mortality, *HCT-CI* Hematopoietic Cell Transplantation-specific Comorbidity Index, *EBMT* European Society for Blood and Marrow Transplantation, *rDRI* revised Disease Risk Index, *KPS* Karnofsky Performance Scale, *BMI* body mass index, ^5^ one unit are 5 ml/min/kg, ^10^ one unit are 10%.

*p ≤ 0.05; **p ≤ 0.01.

^a^Reference group = female.

^b^Reference group = CR/chronic phase.

^c^Reference group = reduced conditioning protocol.

^d^Reference group = low risk, values of intermediate and high risk group are in relation to the low risk group.

^e^Reference group = low-intermediate risk, values of high risk and very high risk group are in relation to the low-intermediate risk group.

^f^Deviation from reference values.

**Table 4:** Fine & Gray model analyses—association of pre-transplant variables with NRM.

	SHR	CI 95%		*P* value
		Lower	Upper	
Univariable analyses				
VO2peak [ml/min/kg]^5^	0.998	0.894	1.114	0.975
Gender^a^	0.173	0.022	1.354	0.095
Age [years]	1.023	0.975	1.074	0.353
Remission^b^	0.515	0.163	1.623	0.257
Conditioning^c^	3.548	0.504	24.959	0.203
rPAM score	1.050	0.952	1.157	0.331
HCT-CI^d^				
Intermediate risk	0.565	0.096	3.325	0.528
High risk	1.997	0.546	7.311	0.296
EBMT score^d^				
Intermediate risk	0.546	0.114	2.603	0.865
High risk	0.866	0.165	4.556	0.865
rDRI^e^				
High risk	0.320	0.092	1.110	0.073
Very high risk	0.076	0.009	0.641	0.018^*^
KPS < 90%	1.430	0.382	5.352	0.595
BMI [kg/m²]	0.990	0.864	1.134	0.886
Fat Mass [%]^10^	0.974	0.965	0.983	<0.001^**^
Lean Body Mass [%]^10^	0.976	0.967	0.985	<0.001^**^
Body Cell Mass [%]^10^	0.975	0.966	0.984	<0.001^**^
Phase angle standardized^f^	1.110	0.630	1.956	0.718
Total energy consumption during exercises/week [kcal]	0.995	0.984	1.005	0.331
Quality of life [%]	1.014	0.983	1.047	0.378
Physical functioning [%]	1.013	0.990	1.036	0.282
Multivariable analysis				
VO2peak [ml/min/kg]^5^	1.070	0.949	1.207	0.271
Gender^a^	0.064	0.008	0.530	0.011^*^
BMI [kg/m²]	0.945	0.788	1.133	0.541
Body cell mass [%]^10^	1.002	0.981	1.024	0.835
rDRI^d^				
High risk	0.088	0.027	0.281	<0.001^**^
Very high risk	0.018	0.003	0.098	<0.001^**^
Conditioning^c^	6.555	0.814	52.777	0.077

*SHR* sub-distribution hazard ratio, *CI* confidence interval, *VO2peak* peak oxygen consumption, *rPAM* revised Pretransplant Assessment of Mortality, *HCT-CI* Hematopoietic Cell Transplantation-specific Comorbidity Index, *EBMT* European Society for Blood and Marrow Transplantation, *rDRI* revised Disease Risk Index, *KPS* Karnofsky Performance Scale, *BMI* body mass index, ^5^ one unit are 5 ml/min/kg, ^10^ one unit are 10%.

*p ≤ 0.05; **p ≤ 0.01.

^a^Reference group = female.

^b^Reference group = CR/chronic phase.

^c^Reference group = reduced conditioning protocol.

^d^Reference group = low risk, values of intermediate and high risk group are in relation to the low risk group.

^e^Reference group = low-intermediate risk, values of high risk and very high risk group are in relation to the low-intermediate risk group.

^f^Deviation from reference values.

### Physical conditioning

Most of our patients (88%) performed CPET till exhaustion quantified by RER ≥ 1.1 or a heart rate ≥ 85% of the age predicted maximum. We detected no difference between 2yrS and 2yrD across experienced and measured exhaustion—in the median, patients reported the level of effort as “very hard” and attained 89% of the maximum predicted heart rate. The VO2peak and maximum reached watt were also comparable. In median all patients reached 120 W (60–280 W) and a VO2peak in relation to body weight of 20 ml/min/kg (12.1–44.8 ml/min/kg). Differences in body composition were only noted in the amount of FM with 2yrS showing a greater FM (median 27% (9–42%); 24% (9–32%), *p* = 0.026). Energy consumption per week and KPS were also comparable (energy consumption/week: 2yrsS 9126 kcal, range 2771–27881 kcal; 2yrD 8060 kcal, range 1965–19488 kcal, *p* = 0.343; KPS: 2yrS and 2rsD 90%, range 70–100%, *p* = 0.375). Detailed descriptions of physical conditioning are found in Table 5.

**Table 5:** Physical conditioning.

	All *N* = 59	2yrsS *N* = 43	2yrsD *N* = 16	*P* value
BMI [kg/m²]^a^	25 (19–33)	25 (19–33)	25 (19–31)	0.653
Fat Mass [% of body weight]^a^	26 (9–42)	27 (9–42)	24 (9–32)	0.026^*^
Lean Body Mass [% of body weight]^a^	74 (56–91)	73 (58–91)	77 (69–91)	0.137
Body Cell Mass [% of body weight]^a^	35 (27–48)	35 (27–48)	38 (30–48)	0.598
Standardized phase angle [°]^a,b^	−1.2 (−4.7–1.4)	−1.1 (−4.7–1.4)	−1.4 (−2.7–0.7)	0.894
Cardiopulmonary exercise test				
VO2peak [ml/min/kg]^a^	20 (12.1–44.8)	20.8 (12–34)	18.9 (12.7–44.8)	0.515
Pmax [W]^a^	120 (60–280)	120 (60–240)	120 (70–280)	0.392
RER^a^	1.1 (1–1.3)	1.1 (1–1.3)	1.1 (1–1.3)	0.913
RPE^a^	17 (13–20)	17 (14–20)	17 (13–20)	0.603
Heart rate % of age predicted maximum^a^	89 (65–109)	89 (70–104)	90 (65–109)	0.785
Total energy consumption during exercises/week [kcal]^a^	8425 (1965–27,880)	9126 (2771–27,881)	8060 (1965–19,488)	0.343
Quality of life [%]	58.3 (16.7–91.7)	58.3 (16.7–91.7)	66.7 (25–91.7)	0.343
Physical functioning [%]	80 (20–100)	80 (20–100)	80 (53.3–100)	0.983

*2yrsS* two-year-survivor, *2yrsD* two-year-decedents, *BMI* body mass index, *VO2peak* peak oxygen consumption, *Pmax* maximum power, *RER* respiratory exchange ratio, *RPE* received perception of exertion.

*p ≤ 0.05.

^a^Median (range).

^b^Deviation from standardized values.

### Risk scores

Risk assessments relying on the EBMT score and the rDRI revealed most patients (61% and 54%) carried an intermediate risk. In turn, using the HCT-CI resulted in the same number of patients having a low, intermediate or high risk. There were no differences between 2yrS and 2yrD in their allocation to different risk groups (EBMT score *p* = 0.768; HCT-CI *p* = 0.550, rDRI *p* = 0.139) or the median rPAM score (*p* = 0.192).

## Discussion

The aim of the present analysis was to examine the explanatory power of pre-alloHCT VO2peak to predict OS and NRM two years after transplantation and to compare this risk prediction to that of commonly-used risk scores. We hypothesized that assessing VO2peak would improve the present risk prediction methods and thus precise post-alloHCT prognosis. Contrary to our assumptions, no association of VO2peak to OS or NRM was found. Only the male gender and the rDRI were associated with OS and NRM two years after transplantation. In univariable analyses, body composition showed an association with OS and NRM, but this could not be confirmed in multivariable models.

Our results stand in contrast to Wood et al. [20] and Kelsey et al. [19], who showed that OS and NRM are partly predictable via the pre-alloHCT VO2peak. However, comparing their patient groups to ours reveals remarkable differences in the numbers of deceased patients. While our mortality rate in this study was only 27% 24 months after transplantation (24 month), Wood et al. [20] reported 37% after 100 days (~3 month) and Kelsey et al. [19] 52% after 25 months. We speculate that our study population was in better health and more physically fit. Compared to Wood el al. [20] our patients suffered from fewer comorbidities (HCT-CI ≥ 3 of 37% vs. 50%) and demonstrated a higher physical performance level, i.e., greater values in maximum reached power during CPET (120 W vs. 47.5 W). However, compared to healthy individuals’ reference values, our study population’s VO2peak was below the 5% percentile [27, 33] indicating that our patients were not especially fit. Furthermore, Kelsey et al. [19] reported even higher VO2peak values and maximum workload during CPET. Nevertheless, CPET-protocol differences can lead to diverging results [34, 35], which can hamper the comparability of patients’ physical capacity.

The association of rDRI with OS we found reflects the assumption that disease-specific determinants can provide best prognosis [36, 37]. Accordingly, the risk of NRM predicted by the rDRI is lower. In general, pre-transplant disease features are more likely to predict relapse mortality, while patient-specific characteristics possess greater validity for transplant-related mortality [4]. A high rDRI risk group means a high tumor burden and therefore patients’ organism is already weakened before alloHCT. This increases the mortality risk [38] which is also indicated by the association of remission status and OS we found in univariable analysis. Shouval et al. [5] described only the random to moderate reliability of pre-transplant risk scores. This is in line with our findings regarding to the remaining scores. The cohorts used for the development of the scores significantly influence the explanatory power. This leads to a better prediction of the rPAM score and HCT-CI for patients undergoing myeloablative conditioning therapy in contrast to patients receiving reduced conditioning protocols [5]. Since 75% of our sample received reduced intensity conditioning, this could be a reason for the missing association between those risk scores and OS as well as NRM.

An explanation why male patients had a higher mortality risk cannot be given. So far it could only be shown that male patients are more affected by complications when they receive female donor cells [39]. But this donor-recipient constellation only existed in four cases.

In univariable analyses higher lean body mass, body cell mass, and fat mass were shown to have an association with reduced mortality risk 2 years after transplantation. Since this correlations could not be confirmed in multivariable analyses, other variables than body composition seem to be decisive for survival of our sample. Nevertheless, in other investigations the pre-transplant loss of body weight was associated with a higher NRM and shorter OS [40]. Furthermore, a low proportion of fat mass in conjunction with low muscle mass before intense medical treatment can raise the mortality risk for cancer patients [41, 42]. Thus, having the appropriate body composition and standard BMI are essential to resisting the treatment-induced loss of body mass, and to withstanding drug toxicity during alloHCT [23, 24]. We assume that not adjusting BMI for gender and age and categorizing values for analysis could have led to the missing association [23]. In addition, infusions may have influenced the measurement of body composition via bioelectrical impedance analysis [43].

For a comprehensive explanation of our results, aspects of statistical analysis must also be taken into account. We provided a suitable sample size (*N* = 59) and observed 16 events (deaths). As the statistical power crucially depends on the number of events, only large prognostic-factor effects are detectable as revealing statistical significance. Thus, the low number of events is a potential weakness in our analysis. However, as comparable investigations [19, 20] did not report the quality of their statistical analysis, it is difficult to classify both their and our results.

The present survival analysis is limited by the fact that our data were analyzed retrospectively and retrieved from a randomized controlled trial that enforced stringent exclusion criteria. That randomized controlled trial was not designed to predict clinical outcomes. Furthermore, collecting data retrospectively can lead to failures to capture relevant data that might not have been recorded.

To best of our knowledge, we are the first to investigate the predictive power of physical conditioning and commonly-used risk scores in relation to survival time within one sample. We could only confirm that the rDRI and male gender predict survival after alloHCT. However, unlike physical performance, these variables cannot be positively influenced and do not reflect all individual preconditions of each patient. Considering the growing numbers of increasingly elderly and frail alloHCT recipients, our results underline the need for further research to identify an all-encompassing and meaningful examination method for predicting patients’ pre-alloHCT risk.
